# Depth of emotional experiencing and outcome in therapy with young people

**DOI:** 10.1111/papt.12537

**Published:** 2024-06-12

**Authors:** Darcy Geyer, Virginia Lam, Hannah Gilbert, Mick Cooper

**Affiliations:** ^1^ University of Roehampton London UK; ^2^ University of Roehampton (Now at the Compassionate Mind Foundation) London UK

**Keywords:** adolescence, counselling, emotional expression, emotion‐focused therapy, experiencing, process‐outcome research

## Abstract

**Objectives:**

To analyse the relationship between depth of emotional experiencing and outcomes in young people. We also wanted to understand whether ‘early’ or ‘working’ depth of emotional experiencing was most predictive of outcomes, and how these compared against alliance effects.

**Design:**

Hierarchical linear regression analysis of data from a study of school‐based humanistic counselling (SBHC).

**Methods:**

Data from 60 young people were used for the study across 15 schools: mean age 13.7 years old (range: 13–16); 67% female; 52% from Black, mixed or non‐white ethnicities. Depth of emotional experiencing was rated using the Client Experiencing Scale at session 1 (*early EXP*) and session 6 (*working EXP*). The dependent variable was changes in psychological distress from baseline to 12‐week follow‐up, as assessed by the Young Person's CORE.

**Results:**

In our final model, working EXP accounted for 11.6% of the variance in YP‐CORE change scores after baseline YP‐CORE scores were taken into account. Early EXP and working alliance were not predictive of benefit. Sensitivity analyses indicated that working EXP was associated with benefits across a range of indicators.

**Conclusions:**

Our findings show, for the first time, that depth of emotional experiencing has a significant and sizeable association with outcomes in therapy for young people. This is consistent with emerging evidence from the adult field. It suggests that practitioners working with young people should monitor the depth of emotional experiencing and foster methods for supporting its development.

**Practitioner points**

- Practitioners should be familiar with, and monitor, depth of emotional experiencing in their therapeutic work with young people.
- Practitioners should consider acquiring—or extending—skills in methods for deepening young people’s emotional experiencing in therapy, such as those from emotion‐focused therapy.

## INTRODUCTION

Globally, one in seven 10–19‐year‐olds meet the criteria for a mental disorder, with evidence that prevalence rates in children and young people (CYP) are increasing (Sadler et al., 2018; World Health Organization, 2021). Childhood disorders often continue into adulthood and can have longstanding social and economic consequences (Chen et al., 2006).

Despite the proliferation of evidence for effective psychotherapies, there has not been commensurate growth in the understanding of key change mechanisms of interventions (Carey, 2011; Kazdin, 2009; Schmidt & Schimmelmann, 2015). This lack of knowledge is particularly evident in the field of child and adolescent psychotherapy. Further advances, exploring a breadth of therapeutic factors and processes, are needed to address the complex and multifaceted nature of change mechanisms in CYP (Gilbert & Kirby, 2019; Missirlian et al., 2005; Norcross & Lambert, 2019) and to provide the basis for novel treatments.

*Emotional experiencing* has been recognised as an important feature of psychotherapeutic change across a range of modalities, most notably the humanistic tradition (Gendlin, 1996; Rogers, 1951, 1959). For Rogers, the goal of therapy was to help clients articulate, accept and transform their emotions (Greenberg & Watson, 2006; Rogers, 1959). *Depth* of this emotional experiencing can be defined as ‘the degree to which clients engage in and explore their feelings moment‐by‐moment as part of the process of personal meaning‐making’ (Pascual‐Leone & Yeryomenko, 2017, p. 2).

Depth of emotional experiencing has been operationalised by the Client Experiencing Scale (EXP; Klein et al., 1969): one of the most studied and validated observational measures in adult psychotherapy research (Harrington et al., 2021; Hendricks, 2009; Pascual‐Leone et al., 2016; Pascual‐Leone & Yeryomenko, 2017). This 7‐point scale evaluates a client's emotional and cognitive involvement in the therapeutic process. Typically, EXP is measured in terms of *modal* EXP, an index of average observed process, or *peak* EXP, the upper limit of the client process (Klein et al., 1986).

Within the adult literature, across therapeutic orientations, higher EXP is predictive of more successful outcomes (Auszra et al., 2013; Elliott et al., 2013; Goldman et al., 2005; Harrington et al., 2021; Hendricks, 2009; Pascual‐Leone et al., 2016; Pos et al., 2003; Whelton, 2004). For instance, Watson and Bedard (2006) examined EXP in relation to outcomes in emotion‐focused therapy (EFT) and cognitive‐behavioural therapy (CBT) for 40 clients with depression. For both therapeutic approaches, good outcomes were associated with higher modal and peak EXP. A meta‐analysis of 10 studies including 406 clients found that—regardless of treatment focus, approach, or phase—EXP significantly predicted treatment outcomes (*r* = −.25; Pascual‐Leone & Yeryomenko, 2017). Pascual‐Leone and Yeryomenko concluded that depth of emotional experiencing is ‘the most promising client process predictor of outcome’ (p. 654). Consistent with these findings, Peluso and Freund (2019), in their meta‐analysis of client emotional expression and treatment outcomes (*k* = 42 studies), found a medium‐to‐large effect size of *r* = .40, 95% CI [0.32, 0.48]. However, these findings must be treated with caution given the emotion‐focused allegiance of many researchers (Luborsky et al., 1999). Studies also tend to focus on populations with depression and, thus, may not be generalisable to wider clinical groups (Barker et al., 2016).

In adults, there is some evidence to suggest that depth of emotional experiencing is predictive of outcomes over and above the therapeutic alliance. Pos et al. (2003) measured modal EXP and alliance in relation to psychotherapeutic improvement of 34 adult clients receiving short‐term humanistic‐experiential therapies for depression. They found that EXP was the sole predictor of gains in self‐esteem and reductions in interpersonal distress and depressive symptoms. Similarly, Goldman et al. (2005) found that increases in EXP across therapy predicted reduced distress symptoms and increased self‐esteem, accounting for variance over and above early EXP and alliance. Subsequent research found that alliance only indirectly contributed to outcomes through supporting increases in EXP (Malin & Pos, 2015; Pos et al., 2009).

To date, there is no published empirical research investigating EXP and outcomes among CYP. Within the EXP manual, Klein et al. (1969) refer to an unpublished doctoral dissertation that used the EXP in a study examining the effectiveness of counselling with institutionalised children (Halpern, 1966 as cited in Klein et al., 1969). However, the method and results of this study are unclear, and there is a lack of adequate peer review. The principal aim of the present study, therefore, is to examine, for the first time, whether depth of emotional experiencing predicts outcomes in young people. We were also interested in exploring whether early or working EXP acts as the stronger predictor of outcomes, and how the association between outcomes and depth of emotional experiencing compares against the alliance–outcome association.

## METHOD

### Design

Hierarchical linear regression modelling was used to analyse the contribution that EXP ratings made to reductions in psychological distress. Data came from the Effectiveness and Cost‐effectiveness Trial of Humanistic Counselling in Schools (ETHOS; Cooper et al., 2021). ETHOS is a randomised control trial, supported by the Economic and Social Research Council [grant reference ES/M011933/1], comparing the clinical and cost‐effectiveness of school‐based humanistic counselling (SBHC) plus pastoral care against pastoral care alone. Ethical approval for ETHOS was granted by the University Ethics Committee of the University of Roehampton in 2016, with trial registration number ISRCTN10460622.

### Participants

Participants for the ETHOS trial were recruited between 29 September 2016 and 8 February 2018 from 18 secondary schools in the Greater London area. All schools were state‐funded: 11 academies, six community schools and one foundation school. The mean number of pupils per school was 900 (SD = 226.10, range = 445–1489). Five of the schools were faith schools (Church of England), and five were single‐sex schools (three female). Seven of the schools (38.9%) were in the most deprived Index of Multiple Deprivation (IMD) quintile, with a further three (16.7%) in the second lowest quintile.

Recruitment was through the schools' pastoral care teams. They were briefed on the trial and, as a pre‐screening stage, asked to identify potentially eligible young people. If young people expressed interest, their parents or carers were contacted by a member of the pastoral care team to provide written consent. Young people were then referred for assessment by a member of the research team who formally assessed their eligibility and invited them to provide written assent.

Eligibility for the ETHOS trial required that participants were aged 13–16 years old and experiencing moderate to severe levels of emotional symptoms (indicated by a score of 5 or higher on the Emotional Symptoms subscale of the self‐report Strengths and Difficulties Questionnaire, SDQ‐ES, range 0–10). Participants also required an estimated English reading age of at least 13 years, wanted to participate in counselling, had a school attendance record of 85% or more (to increase likelihood of attending testing meetings) and were not currently in receipt of another therapeutic intervention. Exclusion criteria were incapable of providing informed consent for counselling, planning to leave the school within the academic year and being deemed at risk of serious harm to self or others.

In total, 329 young people were recruited into the ETHOS trial, with 167 allocated to SBHC (of broadly similar demographics to those in the pastoral care alone arm). Young people were offered up to 10 sessions of therapy. Sessions were scheduled on an approximate weekly basis, with endpoint measures taken at week 12 to allow for missed and rescheduled sessions. *Early EXP* ratings were taken at session 1 and *working EXP* ratings at session 6. We chose session 6 because this was just past the halfway mark for the 10 sessions of therapy: the point at which you people were engaging through to the second half of the intervention.

We screened the 167 cases and selected those that met three criteria. First, there were at least seven sessions. We chose this criterion so that participants engaged with at least one session after the working EXP ratings were taken: meaning that the relationship between working EXP and outcomes would not be wholly retrospective. In addition, seven sessions of therapy have been identified as a minimum for moving into recovery for issues such as depression, anxiety and other disorders (NHS England, 2018, 2020). This filtered out 35 of the 167 cases (20.1%). Our second criterion was that audio recordings for both session 1 and session 6 were available, clearly marked as such, and of sufficient audio quality. Recordings for session 1 did not meet these criteria in 54 cases (41.0% of the remaining 132 cases), and recordings for session 6 did not meet these criteria in a further 12 cases (15.4% of the remaining 78 cases). This left 66 cases, of which six (9.1%) did not meet our third criterion: complete data for all outcome measures.

The demographic characteristics of the 60 remaining young people are listed in Table 1. The mean age of participants was 13.7 years old (SD = 0.66); with 40 female (66.7%) and 20 male (33.3%), Ethnically, 29 of the 60 participants were of White, European and/or British ethnicity (48.3%); and 31 were of Black, Asian or other ethnic minority (51.7%). Seven of the participants had a disability (12%). Scoring bands on the SDQ‐TD indicated that 36 participants in this sample (75%) had ‘high’ or ‘very high’ baseline psychological difficulties. The number of completed SBHC sessions for the 60 participants ranged from 7 to 10, with a mean of 9.1 (SD = 0.98). Compared with ETHOS's clients who were not selected for this analysis, the present sample differed significantly on these demographic characteristics in just two ways. First, there was a higher proportion of male participants (*χ* ^2^[2] = 8.0, *p* = .02). Second, there was a greater number of sessions (*F*[1, 166] = 22.0, *p* < .001)—an expected difference given we only selected young people with seven or more sessions.

**TABLE 1:** Baseline characteristics of study participants and all SBHC participants.

	Study participants (*N* = 60)	All SBHC (*N* = 167)
Gender*		
Female	40 (67%)	127 (76%)
Male	20 (33%)	37 (22%)
Other	0 (0%)	3 (2%)
Age (SD)	13.7 (0.7)	13.7 (0.8)
Number of sessions**	9.1 (1.0)	7.9 (2.6)
Baseline psychological difficulties (SDQ‐TD)		
Close to average	5 (8%)	20 (12%)
Slightly raised	11 (17%)	33 (20%)
High	8 (20%)	22 (13%)
Very high	28 (55%)	87 (52%)
Ethnicity		
White, European and/or British	29 (48%)	92 (55%)
Black, mixed and other	31 (52%)	74 (45%)
Missing		1 (0%)
Disability		
No disability	52 (88%)	142 (85%)
Has a disability	7 (12%)	23 (14%)
Missing	1 (2%)	2 (1%)

*Note*: *p < .05, **p < .01 included in study vs. not included in study.

Abbreviation: SBHC, school‐based humanistic counselling.

### Measures and materials

#### Client Experiencing Scale (EXP)

The Client Experiencing Scale (EXP; Klein et al., 1969) is a measure of depth of emotional experiencing. The scale asks judges to rate this depth on a 1–7 scale, with higher numbers indicating greater depth. The manual has detailed descriptions of the characteristics of expressions at each stage of the scale. Stage 1 expressions are general, impersonal and superficial, revealing no personal information about the client and avoiding addressing feelings. At the other end, the content of stage 7 expressions reveals the client's steady and expanding awareness of their immediately present feelings or internal processes, linking and integrating felt nuances of experience as it occurs, such that each new level of self‐awareness functions as a catalyst for further exploration.

Psychometric evidence for the EXP has demonstrated its inter‐rater reliability (0.76–0.91), retest stability (*r* > .80), and predictive validity for changes in symptoms of depression, trauma or interpersonal and self‐esteem problems (Klein et al., 1986; Pascual‐Leone & Yeryomenko, 2017; Watson & Bedard, 2006).

#### Young Person's CORE (YP‐CORE)

The Young Person's CORE (YP‐CORE) was used as the primary outcome measure for the present analysis. The YP‐CORE is a self‐report measure of psychological distress in young people (Twigg et al., 2009, 2016) and the most commonly used outcome measure in secondary school‐based counselling in the UK (Cooper, 2013). Young people are asked to rate their psychological distress on 10 items using a 5‐point scale (0–4), giving a total score between 0 and 40, with higher scores indicating greater levels of distress. The YP‐CORE measure has been shown to be acceptable to CYP, with a good level of internal consistency (Cronbach's α = 0.85, Twigg et al., 2009), test–retest stability (Pearson's *r* = .76, Twigg et al., 2016) and differentiation between means for clinical and non‐clinical samples (19.0 [SD = 7.5] and 9.4 [SD = 7.3], respectively, Twigg et al., 2016).

#### Working Alliance Inventory short form (WAI‐S)

The WAI‐S is a 12‐item measure, adapted from the Working Alliance Inventory (WAI, Horvath & Greenberg, 1989) which assesses the collaborative and affective bond within the therapeutic relationship (Tracey & Kokotovic, 1989). Like the WAI, the WAI‐S is based on Bordin's (1979) tripartite model of the working alliance and consists of three 4‐item subscales: agreement on the goals of the therapeutic relationship (Goal subscale), collaboration on the tasks needed to achieve these goals (Task subscale) and the quality of the therapeutic relationship (Bond subscale). Respondents are asked to rate each item in terms of how much it describes how they think or feel about their counsellor. For instance, ‘I feel that [mentally insert name of counsellor] appreciates me’ (Item 7, Bond subscale). The total WAI‐S score, consisting of the summed scores from these three subscales, ranges from 12 to 84 (higher scores indicating greater alliance) and was used as the measure of alliance for the present study. The WAI‐S is the most used alliance measure with adolescents and has demonstrated good internal consistency within youth samples (Cronbach's α = 0.94, Capaldi et al., 2016).

#### Secondary measures

We used a range of secondary outcome measures, all of which have evidence of reliability and validity. We assessed psychological difficulties using the self‐report Strengths and Difficulties Questionnaire Total Difficulties (SDQ‐TD) (Goodman, 2001), symptoms of depression and anxiety using the Revised Children's Anxiety and Depression Scale–Short Version (RCADS‐SV) (Ebesutani et al., 2012), self‐esteem using the Rosenberg Self‐esteem Scale (RSES) (Rosenberg, 1965), well‐being using the Warwick‐Edinburgh Mental Well‐being Scale (WEMWBS) (Tennant et al., 2007), personal goals using the Goal‐Based Outcome Record Sheet (GBORS) (Law & Jacob, 2015) and satisfaction with treatment provision using the satisfaction with care subscale of the Experience of Service Questionnaire (ESQ) (Attride‐Stirling, 2003; Brown et al., 2014).

### Procedure

Young people entering the ETHOS trials were randomly assigned (1:1) to either SBHC plus usual pastoral care or usual pastoral care alone. Allocation was concealed and undertaken centrally via remote access to a secure randomization procedure.

SBHC is a manualised form of humanistic therapy based on evidence‐based competences for humanistic counselling with young people aged 11–18 years (Kirkbride, 2016). SBHC assumes that distressed young people have the capacity to address their difficulties if they can explore them with an empathic, supportive and trustworthy counsellor. SBHC counsellors use a range of techniques, including active listening, empathic reflections and inviting young people to express underlying emotions and needs. Sessions were delivered on an individual, face‐to‐face basis and lasted 45–60 min. They were scheduled weekly over a period of up to 10 school weeks, with young people able to terminate counselling prior to this time point. Counsellors were instructed to audio record sessions on a digital device (video recording was not considered feasible in a school setting).

Across the 60 young people in this study, SBHC was delivered by a pool of 12 counsellors across 15 schools (with nine counsellors working in one school each and three working in two schools). The counsellors had between one and nine clients each, with an average of five clients per counsellor. Nine of the counsellors were female and three male, with a mean age of 45.0 years old (SD = 10.0). Eight of the counsellors were of a white British ethnicity and four were of a black Caribbean or African ethnicity. All counsellors were qualified to diploma level (at least a 2‐year, part‐time training) and had been qualified for an average of 5.0 years (SD = 4.2).

The counsellors were instructed to adhere to a SBHC manual, developed for the trial. They received, at minimum, 4 days of group training, with an additional day's training in the research protocols. Adherence to SBHC was assessed by two independent auditors using a young person's adapted version of the Person Centred and Experiential Psychotherapy Rating Scale (PCEPS‐YP). The mean counsellor adherence rating was 4.6 on the 6‐point PCEPS‐YP (SD = 0.4), with all counsellors exceeding the pre‐defined adherence cut‐point, based on the PCEPS literature, of 3.5.

YP‐CORE scores and all secondary outcomes (excepting the ESQ) were measured at baseline assessment, 6 weeks post‐baseline and 12 weeks post‐baseline. The ESQ, as a satisfaction with care measure, was assessed at 12 weeks post‐baseline only. We also used follow‐up scores for YP‐CORE at 24 weeks post‐baseline. WAI‐S scores were measured at 6 weeks post‐baseline. Testers, at all time points, were blind to participants' allocations.

For each of the 60 participants in the present analysis, EXP ratings were conducted in two sessions: session 1 (*early EXP*) and session 6 (*working EXP*). This gave 120 EXP ratings in total. These ratings were conducted by the first author on full session audio recordings, blind both to outcomes and session numbers. Ratings were based, as per the manual (Klein et al., 1969), on the client's verbal content and not other variables, such as the therapist's verbal content, the timing, context, or purpose of the session. The first rater (as with the second rater) listened to each session in its entirety, noting an EXP rating for all client statements. The rater then recorded on a database the peak EXP score: that is, the young person's most attained level of emotional processing within the session (Klein et al., 1986). Peak EXP has shown similar outcomes to modal EXP, with peak moments in the working phase hypothesised to ‘lead to the consistent changes in emotional processing later in therapy that clients may need’ (Pos et al., 2009; p. 1063), Thus, we felt that peak EXP particularly suited our primary outcome: changes in psychological distress.

To assess retest stability, 60 of the recordings (30 from Session 1 and 30 from Session 6) were randomly selected and re‐rated blind by the first author, 2 weeks after the initial rating. Cohen's kappas (Cohen, 1960) indicated substantial agreements between original and subsequent ratings: *κ* = 0.80 for Session 1 ratings, *κ* = 0.87 for Session 6 ratings.

To assess inter‐rater reliability, 30 sessions were selected randomly for independent re‐rating by a second rater, a Master's trainee in CYP counselling, who was also blind to outcomes and session number. Initial cases were used by this second rater to practise the use of the EXP scale, with instruction from the first author, before the second rater rated the remaining cases independently. Across the 30 sessions, Cohen's kappas indicated moderate and substantial levels of inter‐rater reliability for early EXP ratings, *κ* = 0.55, and for working EXP ratings, *κ* = 0.75, respectively. For our analyses, to maintain consistency across ratings, we used just the original ratings of the first author.

### Analysis

Although clients were clustered within counsellors and schools, the relatively low number of clients per counsellor meant that we did not attempt a multilevel analysis. All data were analysed using the Statistical Package for the Social Sciences (SPSS) v28.0.0.1. As exploratory analyses, we examined descriptive data for early and working EXP ratings and correlations with key variables. The principal inferential test consisted of a hierarchical linear regression analysis to examine the contribution of early and working EXP ratings on outcomes. The dependent variable was reductions in psychological distress, as measured on the YP‐CORE from baseline to the week 12 follow‐up. We entered our independent variables in blocks to reflect their approximate temporal ordering in the counselling. We first entered baseline YP‐CORE scores and demographic characteristics (age, gender, ethnicity and disability status). Then we entered early EXP ratings (session 1). We then added working alliance scores assessed at the 6‐week testing meeting. Finally, we entered working EXP ratings from session 6.

#### Sensitivity analyses

We conducted a range of post hoc sensitivity analyses to assess the robustness of the hierarchical linear regression findings. First, to control for the possible imprecisions of a temporally based block entry approach, we analysed the data entering all variables together using stepwise regression. Second, to see whether our effects were limited to psychological distress as measured by the YP‐CORE, we replaced the dependent variable in our stepwise regression model with a range of secondary 0–12 week change scores (using the respective baseline scores, where relevant) and number of sessions attended. Third, we replaced our 0–12‐week YP‐CORE change scores with 6–12‐week change scores (entering, as a baseline, 6‐week scores), to control for the possibility that pre‐6‐week change might account for an association between working EXP and outcomes. We also used as our dependent variable 0–24‐week YP‐CORE scores, to consider longer term effects. Finally, in our original regression model, we replaced our session 1 and session 6 EXP scores with a ‘change in EXP’ variable (standardised residuals from session 6 regressed on session 1), to assess whether in‐person increases in EXP—as opposed to between‐person differences in EXP—were responsible for any differences in YP‐CORE change scores.

## RESULTS

### Preliminary analyses

The mean early EXP rating was 4.3 (SD = 0.79), and the mean working EXP rating was 4.90 (SD = 1.41). Ratings were normally distributed with no evidence of significant skewness or kurtosis. The correlation (*r*) between early EXP and working EXP was .67 (95% CI = 0.50, 0.79). A paired *t*‐test indicated that there was a significant increase of EXP ratings from early EXP to working EXP of 0.60 points on the 1–7 scale, *t*(59) = 4.38, *p* < .001, Hedges' *g* = 0.56 (95% CI = 0.29, 0.82).

**FIGURE 1:**
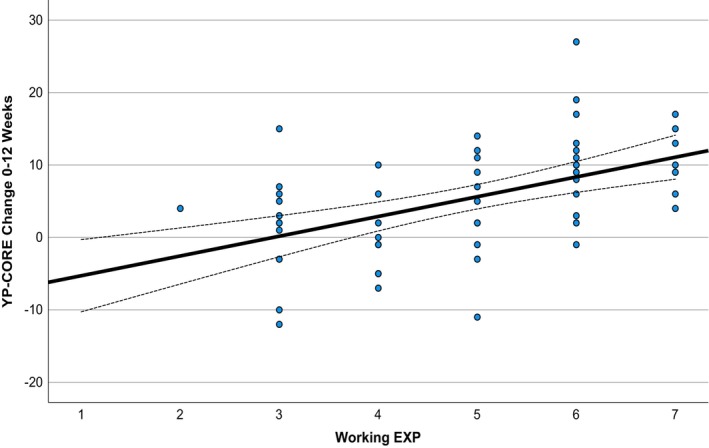
Scatterplot of working EXP ratings against changes in YP‐CORE scores from baseline to 12 weeks. YP‐CORE, Young Person's CORE.

Early EXP and working EXP scores did not vary significantly across age, gender, ethnicity or disability status. They also did not correlate significantly with working alliance scores. Changes in YP‐CORE scores from baseline to 12‐week follow‐up did not correlate significantly with early EXP ratings, *r* = .14 (95% CI = −0.12, 0.38). However, they did correlate significantly with working EXP ratings, *r* = 0.51 (95% CI = 0.30, 0.68) (see Figure 1).

### Hierarchical linear regression

As expected, baseline YP‐CORE scores were a significant predictor of YP‐CORE change 0–12 weeks: *b* = 0.51, b* = 0.42, *t* = 8.1, *p* < .001, *R* ^2^ = .24. Model fit was not significantly enhanced by the addition of age (*t* = −1.59, *p* = .11), gender (*t* = −1.01, *p* = .31), ethnicity (*t* = 0.11, *p* = .91) or disability status (*t* = −1.43, *p* = .15). These demographic variables were excluded from further analysis. Early EXP ratings also did not significantly add to model fit (*t* = 0.84, *p* = .41) and were excluded. Adding WAI‐S scores contributed significantly to model fit, *b* = 0.09, b* = 0.17, *t* = 2.31, *p* = .02, Δ*R* ^2^ = .03. Finally, the addition of working EXP ratings added significantly to model fit, *b* = 1.88, b* = 0.35, *t* = 3.09, *p* = .003, Δ*R* ^2^ = .10; with WAI‐S scores now no longer significantly contributing to the model (*t* = 1.26, *p* = .21). Hence, our final linear regression model consisted of YP‐CORE baseline scores and working EXP ratings, accounting for 35.4% of variance in YP‐CORE changes from 0 to 12 weeks. Each additional point of baseline YP‐CORE score was associated with a 0.4 increase in YP‐CORE change, and each additional point of working EXP ratings was associated with a 2.0 increase in YP‐CORE change. Working EXP accounted for 11.6% of variance in YP‐CORE change scores after baseline YP‐CORE scores were taken into account.

### Sensitivity analyses

Our stepwise regression, with all variables entered in the same block, produced two models: the first, with just working EXP ratings, accounting for 26% of variance overall. A second model, with both working EXP ratings and baseline YP‐CORE scores, accounted for 37.4% of variance overall. All other variables were excluded.

Using the alternative change scores as our dependent variables, we found that working EXP continued to make a significant contribution to six of our seven final models: SDQ psychological difficulties (b* = 0.25, *t* = 2.06, *p* = .04), RCADS depression and anxiety (b* = 0.33, *t* = 2.68, *p* = .01), RSES self‐esteem (b* = 0.34, *t* = 2.69, *p* = .01), WEMWBS well‐being (b* = 0.40, *t* = 4.03, *p* < .001), GBOR goal attainment (b* = 0.29, *t* = 2.27, *p* = .03) and number of sessions (b* = 0.30, *t* = 2.40, *p* = .02). The one exception was ESQ satisfaction with care, which working EXP did not significantly predict (b* = 0.10, *t* = 0.77, *p* = .45). Early EXP contributed to just one final model, ESQ satisfaction with care (b* = 0.26, *t* = 2.63, *p* = .01); and WAI‐S working alliance contributed to two final models: ESQ satisfaction with care (b* = 0.67, *t* = 6.91, *p* < .001) and WEMWBS well‐being (b* = 0.24, *t* = 2.41, *p* = .019).

When YP‐CORE change scores from 6 to 12 weeks were used as the dependent variable, we found that working EXP remained a significant predictor in the final model (b* = 0.44, *t* = 2.68, *p* = .01) (along with 6‐week YP‐CORE scores). Baseline EXP and WAI‐S scores were not significant. However, using 0–24 week YP‐CORE change scores as dependent variables, neither working EXP nor early EXP (nor working alliance) made significant contributions to model fit.

EXP change scores, like working EXP ratings, added significantly to model fit in predicting YP‐CORE change scores, over and above baseline YP‐CORE scores: *b* = 3.28, b* = 0.43, *t* = 3.88, *p* < .001, Δ*R* ^2^ = .15. In total, this model accounted for 38.5% of variance in YP‐CORE change scores: 3.1% more variance than when working EXP scores were used alone.

## DISCUSSION

The principal aim of the present study was to examine whether the depth of emotional experiencing predicted outcomes in therapeutic work with young people. For the first time, we established that there was a significant association, contributing approximately 12% of the variance in outcome scores once baseline scores were taken into account. This is highly consistent with the results of both Pascual‐Leone and Yeryomenko's (2017) and Peluso and Freund's (2019) meta‐analyses, suggesting that depth of experiencing is one of the most promising client process predictors of outcome for youth clients as well as adults.

With respect to the effects of early EXP, our study supports the findings of Missirlian et al. (2005) that clients' pre‐therapeutic capacities for emotional experiencing do not predict outcomes. Our sensitivity analysis of change scores also indicated that it was within‐person increases in emotional experiencing, rather than between‐person baseline differences, that were associated with therapeutic change. This is somewhat inconsistent with the findings of studies which suggest that different pre‐therapeutic capacities for emotional experiencing predict subsequent improvements in psychological functioning (Goldman et al., 2005; Hendricks, 2002; Pos et al., 2003). It is also inconsistent with the large body of theory suggesting that clients' capacities for experiential processing are key predictors of improvement (e.g. Gendlin, 1996). More broadly, this result is at odds with a view of the client as the active change agent in therapy (e.g. Bohart & Tallman, 1999). Instead, these findings suggest that young people may not need to start therapy with the capacity for in‐depth processing to obtain favourable outcomes. Rather, this is something that can evolve through the therapeutic process. In line with adult literature, therefore, there is evidence that young people can learn to deepen their capacity for emotional experiencing through therapy (Hendricks, 2009; Pascual‐Leone & Yeryomenko, 2017).

We found, almost consistently, that working EXP was a stronger predictor of outcomes than alliance. This parallels the majority of existing research comparing both variables in adult clients (Goldman et al., 2005; Hendricks, 2009; Malin & Pos, 2015; Pos et al., 2003, 2009). It also, at the very least, supports Pascual‐Leone and Yeryomenko's (2017) assertion that the EXP is of a similar predictive magnitude to the therapeutic alliance. Indeed, the present findings emphasise the importance of EXP processes, beyond the alliance process, in promoting change (Goldman et al., 2005).

As the present study had relatively high therapeutic alliance ratings on average (M = 64.25, SD = 13.19), it could be argued that this supported increases in EXP, as found by some earlier studies (Fisher et al., 2016; Goldman et al., 2005; Malin & Pos, 2015; Pos et al., 2009; Town et al., 2017). However, some adult research comparing emotional expression in high and low cases of working alliance concluded that emotional experiencing is neither augmented nor inhibited by the level of therapeutic alliance (Iwakabe et al., 2000), though this is challenged by Fisher et al. (2016), whose study showed that higher scores for therapeutic alliance at the end of the first session predicated greater emotional experience in the next session. More recently, Harrington et al. (2021) identified individual differences in the extent to which WAI and EXP contributed to outcomes in adults who received EFT for childhood trauma. Whilst this somewhat parallels our working EXP findings, the present study did not have a baseline measure of therapeutic alliance for comparison. In light of this and given the discrepancies in this area and the complexity of the relationship between WAI and EXP, further research is warranted to investigate high and low therapeutic alliance ratings and how these interrelate with EXP across stages of interventions with CYP.

The strengths of the present study include analysis of data from a standardised, adherence‐monitored intervention, with a range of secondary measures and time points to triangulate principal findings and clients who came from a diverse range of ethnic backgrounds and socio‐economic statuses. The research team also had a relatively high level of equipoise, with no strong allegiance to ‘proving’ that emotional processes were predictive of greater improvements. Indeed, the second author is an established advocate of both relational approaches to therapy and the role of client‐agency in therapeutic outcomes: assumptions which are challenged by the present findings.

An important limitation of our study is that the first author was also the principal rater. While the ratings were conducted blind and closely replicated by a second independent rater, it is still conceivable that these ratings were biased in some way. For instance, the first author might have sensed, from the session 6 recordings, whether or not the client was improving and biased their judgements of emotional experiencing accordingly. That the second rater was trained by the first rater, rather than by an independent researcher, also has the potential to lead to bias. Our reliance on peak EXP ratings is also a significant limitation. Although, in previous studies, these have shown similar outcomes to modal EXP ratings (e.g. Pos et al., 2003, 2009), it would have been ideal to record both forms. However, our finding that working EXP predicted subsequent 6–12 week change corroborates previous findings of peak EXP being a predictor of later change therapy (Pos et al., 2009).

As with all observational studies, the study is limited by the correlational nature of the findings (Janse et al., 2021). It is possible, for instance, that emotional experiencing increased as a result of reduced psychological distress and is, therefore, a by‐product, rather than a cause, of improvement. Again, however, this is mitigated somewhat by our sensitivity analysis, showing that working EXP predicted subsequent change. It is also possible that a third variable, such as increasing motivation for therapy, determined both emotional experiencing and outcomes. The alliance and outcome measures used in the study were all self‐reported, the use of which—particularly with CYP—has been challenged (Edelbrock et al., 1985; McLeod, 2001). Representativeness is limited, as this study consisted of a relatively small, homogenous sample of school students aged 13–15 years, with moderate‐to‐severe levels of psychological distress. Our sample may also not be representative of those attending school counselling with more sessions attended and a higher proportion of males. As our study focused on humanistic counselling, we do not know if these findings are relevant to other therapeutic orientations, and particularly those, such as CBT, that tend to place less emphasis on emotional experiencing in therapy.

Future research would benefit from exploring emotional experiencing and outcomes in a larger and more diverse sample of young people: across a variety of therapeutic orientations, presenting difficulties and contexts. Multiple independent trained raters of EXP would be highly valuable for such studies. Longer‐term outcomes need further investigation. The development of experimental designs for future research would also be very welcome; for instance, comparing the outcomes of youth counsellors trained—or not trained—in emotion‐focused practices. Future research should also explore reasons for different levels of engagement with therapy—whether that may be related to EXP—and associated outcomes.

Despite the limitations of this study and the need for further enquiry, our analysis has produced some compelling, ground‐breaking results in the field of youth therapy. We have established, for the first time, that depth of emotional experiencing is predictive of therapeutic outcomes: accounting for over 10% of outcomes and at a level that consistently dwarves alliance effects. Post hoc analyses suggest that this effect is sustained across a variety of outcomes. If replicated in different contexts, these findings would have major implications for the practice of therapy with young people, suggesting that methods and strategies that help clients access and express emotional experiencing should be at the forefront of practitioners' work. Currently, the British Association for Counselling and Psychotherapy's competences for humanistic counselling with CYP (British Association for Counselling and Psychotherapy, 2019) include ‘Approaches to working with, and making sense of, emotions’ as an optional set of ‘specific’ competences. Replication of our findings would suggest that they may be better placed as more fundamental ‘basic’ competences: essential skills that every counsellor working with young people should be able to apply. The replication of our findings would also support the development of emotion‐focused therapy for young people (e.g. Foroughe, 2023): an integrated approach to therapy which puts emotional evocation and processing at the heart of its therapeutic enterprise.

## AUTHOR CONTRIBUTIONS

**Darcy Geyer:** Conceptualization; methodology; writing – review and editing; data curation; formal analysis; project administration; writing – original draft. **Virginia Lam:** Conceptualization; methodology; writing – review and editing. **Hannah Gilbert:** Conceptualization; methodology; writing – review and editing; data curation. **Mick Cooper:** Conceptualization; methodology; writing – review and editing; data curation; formal analysis; writing – original draft; funding acquisition.

## CONFLICT OF INTEREST STATEMENTS

All authors declare that they have no conflicts of interest.

## Data Availability

Quantitative, participant‐level data (with data dictionary and related documents) for the ETHOS trial are available via the ReShare UK Data Service https://doi.org/10.5255/UKDA‐SN‐853764. Access requires ReShare registration. EXP ratings and qualitative data (including session transcripts) are available from the corresponding author upon request.
